# Mental Health in Pregnant Adolescents: Associations with Family Structure, Educational Continuity, and Marital Status

**DOI:** 10.3390/bs16020221

**Published:** 2026-02-03

**Authors:** Carmen Hernández-Chávez, Reyna Sámano, Gabriela Chico-Barba, Hugo Martínez-Rojano, Colomba Elías-Fernández, Estefania Aguirre-Minutti, Hector Borboa-Olivares, Rosalba Sevilla-Montoya, Yuridia Martínez-Meza, Sandra Martínez-Medina

**Affiliations:** 1 Departamento de Neurobiología del Desarrollo, Instituto Nacional de Perinatología, Mexico City 11000, Mexicosandys65.sm@gmail.com (S.M.-M.); 2 Coordinación de Nutrición y Bioprogramación, Instituto Nacional de Perinatología, Mexico City 11000, Mexico; gabyc3@gmail.com (G.C.-B.); minuttiestefania@gmail.com (E.A.-M.); 3 Escuela Superior de Medicina, Instituto Politécnico Nacional, Mexico City 11340, Mexico; 4 Departamento de Salud, Nutrición y Ciencias de los Alimentos, Universidad Iberoamericana, Mexico City 01219, Mexico; 5 Subdirección de Investigación Clínica, Instituto Nacional de Perinatología, Mexico City 11000, Mexico; 6 Coordinación de Genética y Genómica Humana, Instituto Nacional de Perinatología, Mexico City 11000, Mexico

**Keywords:** prenatal mental health, adolescent pregnancy, sociodemographic factors, marital status, educational attainment, family structure

## Abstract

This study investigated the intricate relationship between sociodemographic factors and mental health indicators among a cohort of pregnant adolescents. We conducted a cross-sectional analysis of 338 primigravidas, aged 11–19 years, systematically collecting sociodemographic data, including age, socioeconomic status, schooling, occupation, marital status, and family structure. Mental health was assessed using validated scales for depressive symptoms (EPDS), state–trait anxiety inventory (STAI), self-esteem (Coopersmith), and perceived stress (PSS-4). A substantial portion of the cohort presented with significant mental health challenges: 33.5% screened positive for depressive symptoms, 18% for state anxiety, 23% for trait anxiety, 67% reported low self-esteem, and 52% experienced high perceived stress. Specifically, working adolescents exhibited markedly higher odds of depressive symptoms (OR 3.516), low self-esteem (OR 1.091), elevated state anxiety (OR 2.803), and increased trait anxiety (OR 2.455). Adolescents living with a partner also showed a greater likelihood of reporting depressive symptoms (OR 1.921), heightened state anxiety (OR 1.772), and increased trait anxiety (OR 2.335). Additionally, lower educational attainment (OR 1.885) and residing in extended family structures (OR 1.894) were associated with diminished self-esteem. These findings underscore the significant influence of occupation, family structure, and marital status on the mental health trajectories of pregnant adolescents. Consequently, promoting educational continuity and fostering greater autonomy in personal life decisions for adolescents could be crucial interventions to improve their emotional well-being during pregnancy.

## 1. Introduction

Adolescent pregnancy is a major public health challenge, particularly in low- and middle-income countries such as Mexico. In 2023, the adolescent fertility rate in Mexico was 61.5 births per 1000 women aged 15–19 years (National Institute of Statistics and Geography INEGI, 2023). While maternal mental health has been widely studied in pregnant women overall (Hodgkinson et al., 2013), the specific mental health needs of pregnant adolescents remain underexplored, despite evidence of their heightened vulnerability during and after pregnancy (Youseflu et al., 2023).

Globally, studies consistently show that pregnant adolescents are more vulnerable to affective disorders, particularly depression and anxiety (Rivera, 2024), stress and low self-esteem than adult women. This increased risk is strongly influenced by social determinants such as marginalization, intimate partner violence, stigma, school dropout, and limited family and social support (Tembo et al., 2022; Tele et al., 2022; Siegel & Brandon, 2014).

In Latin America, and especially in Mexico, structural inequality, limited access to perinatal mental health services, and restrictive social norms further intensify this vulnerability. This is particularly concerning given that adolescence is a developmental stage marked by identity formation and heightened psychological sensitivity (Romeo, 2010), which pregnancy may further exacerbate (ENAPEA, 2024). Recent evidence indicates that 30–44% of pregnant adolescents experience depressive symptoms, with comparable rates of generalized anxiety (Tele et al., 2022; ENAPEA, 2024), as well as low self-esteem and elevated stress levels (Romeo, 2010).

Pregnant adolescents frequently navigate challenging social environments and confront higher rates of unplanned pregnancies (Laurenzi et al., 2020). The dual transition of both adolescence and pregnancy significantly amplifies physical and psychological vulnerability (Romeo, 2010). Consequently, approximately 31.9% of adolescents exhibit anxiety symptoms (Vanderkruik et al., 2021) and higher rates of depression (Eichler et al., 2019), often accompanied by social isolation and strained parental relationships, particularly among those from low socioeconomic backgrounds (Venkatesh et al., 2014).

However, despite the high prevalence of adolescent pregnancy and its well-documented social vulnerability in Mexico (Rivera, 2024; Tembo et al., 2022; Tele et al., 2022; Siegel & Brandon, 2014; ENAPEA, 2024), there is limited empirical evidence that simultaneously examines mental health risk indicators and key sociodemographic determinants in this population. Existing studies often focus on isolated outcomes or lack an integrated analysis of how factors such as family structure, educational continuity, marital status, and occupational roles interact to shape psychological vulnerability among pregnant adolescents in the Mexican context.

Despite the recognized importance of mental health during adolescent pregnancy, the influence of sociodemographic factors remains insufficiently characterized. These factors constitute key contextual determinants of both maternal well-being and child development. To date, few studies have addressed this issue in Latin America, including Mexico (Hodgkinson et al., 2013; Rivera, 2024; Tele et al., 2022; Siegel & Brandon, 2014). Although adolescent pregnancy affects women’s health, the relationship between sociodemographic factors and mental health indicators has been scarcely explored. Accordingly, this study aimed to analyze the association between sociodemographic factors and mental health risk indicators within a cohort of pregnant adolescents, with the goal of identifying vulnerable profiles that may inform targeted screening and preventive strategies in maternal and adolescent healthcare.

## 2. Materials and Methods

### 2.1. Study Population

This cross-sectional study included primigravid adolescents under 19 years of age who received prenatal care and delivered at the Instituto Nacional de Perinatología (INPer) in Mexico City. Participants were drawn from low-income families lacking social security coverage and resided within the metropolitan area of Mexico City. Therefore, the study population is representative of pregnant adolescents receiving public-sector perinatal care in urban, low-resource settings, which constitute a substantial proportion of adolescent pregnancies in Mexico.

Eligibility criteria included singleton pregnancies and absence of prior or gestational psychiatric diagnoses, chronic non-communicable diseases, or regular medication use. Adolescents with substance use disorders, pregnancies resulting from sexual abuse, or cases involving congenital malformations or fetal death were excluded.

The sample size was calculated for an association study (Althubaiti, 2022), aiming to detect statistically meaningful associations between sociodemographic variables and mental health indicators with a 95% confidence level and 80% statistical power (*α* = 0.05, *β* = 0.20). To account for potential non-response, missing data, or incomplete questionnaires, the estimated sample size increased by 15%, resulting in a final target sample of 338 participants.

Of the 400 eligible adolescents invited, 385 agreed to participate (96.3%). Among them, 350 completed the mental health assessments, and 338 had complete data for all study variables and were included in the final analyses. Adolescents with incomplete data did not differ from those included in key sociodemographic characteristics, indicating a low risk of selection bias.

Assent and informed consent were obtained prior to participation. Adolescents younger than 18 years provided written assent, in addition to written informed consent from their parents or legal guardians. Sociodemographic data were collected using a standardized form. All assessments were conducted during the second trimester by trained health professionals in private clinical settings, ensuring participant confidentiality and comfort.

This study utilized data derived from two research protocols, both approved by the INPer institutional committees on Research, Ethics, and Biosafety (registry numbers 2017-1-101 and 2024-1-61); these protocols assessed the same variables within the identical study population and were classified as minimal risk research. Adolescents identified as being at risk for anxiety, depressive symptoms, stress, or low self-esteem were referred to institutional mental health services for appropriate care, in accordance with ethical guidelines. As the study was cross-sectional and all assessments were completed prior to referral, these procedures did not affect the study results.

Data were collected under two independent but methodologically identical research protocols. The first phase was conducted between 2017 and December 2019, followed by a suspension during 2020–2021 due to the COVID-19 pandemic. Data collection resumed in January 2022 and continued through 2024. Both protocols were implemented in the same geographic area and target population, using identical study design, instruments, and data collection procedures; therefore, datasets were harmonized and combined for analysis.

### 2.2. Measures

Mental health indicators were assessed using several validated instruments:

- **Depressive Symptomatology:** Depressive symptoms were measured using the Edinburgh Postnatal Depression Scale (EPDS), a validated 10-item tool with a four-point Likert scale (total scores 0–30) for detecting depressive symptoms during pregnancy and the postpartum period (Bergink et al., 2011). The EPDS has been validated in Mexican adolescent populations. For this analysis, a cutoff score of 13 was utilized for this analysis (Alvarado-Esquivel et al., 2014).
- **Anxiety:** Anxiety was assessed with the State–Trait Anxiety Inventory (STAI), which evaluates both state anxiety (a transient emotional condition) and trait anxiety (a general predisposition to anxiety). Each subscale comprises 20 items rated on a four-point scale. The STAI has been validated for use in pregnant populations (Morales & González, 1990). Cronbach’s alpha coefficients were 0.91 for the state scale and 0.83 for the trait scale (García-Rodríguez & García-Rodríguez, 2021).
- **Perceived Stress:** Perceived stress was evaluated using the Spanish version of the Perceived Stress Scale (PSS-4), which gauges the extent to which individuals perceive their lives as unpredictable or stressful. This four-item scale uses a five-point rating (0–4), yielding total scores from 0 to 16 (Flores-Torres et al., 2021). Scores were categorized into tertiles, with the highest tertile serving as the outcome variable.
- **Self-Esteem:** Self-esteem was measured via the Coopersmith Self-Esteem Inventory, composed of 25 dichotomous (“yes”/“no”) items. Total scores range from 0 to 25, with higher scores indicating greater self-esteem (Cantú et al., 1993). For analytical purposes, scores were multiplied by four to standardize the range from 0 to 100.

### 2.3. Sociodemographic Factors

Socioeconomic status was determined using an eight-question survey developed by the Mexican Association of Market Intelligence and Public Opinion Agencies (AMAI, 2014), which assesses household conditions, purchasing power, and related factors. Although six levels are typically defined, the predominant categories in this study were lower-middle, low, and very low.

Other sociodemographic variables, including educational level, occupation, age, marital status, and family structure, were collected through direct questioning and recorded on standardized forms. Categorical variables were classified as follows:

- **Education:** primary, secondary, or high school.
- **Marital status:** single or partnered/married.
- **Socioeconomic level:** lower middle, low, or very low.
- **Occupation:** student, homemaker, or employed.
- **Family structure:** nuclear (parents and children), extended (parents, children, and other relatives), composite (parent, stepparent, and children), or single parent (one parent and children living together). Family structure was conceptualized as a contextual and relational factor reflecting household composition and caregiving dynamics, capturing culturally relevant living arrangements in our population.

Additionally, chronological age, gynecological age, gestational age at the initiation of prenatal care, and gestational age at assessment were recorded. Gynecological age and partner’s age were explored descriptively but were not included in the regression analyses to avoid overadjustment and multicollinearity with chronological age and marital status. Hours of total nocturnal sleep across the night and early morning, identified a priori as a proximal behavioral factor associated with mental health outcomes, was included as the sole adjustment variable in the analyses.

### 2.4. Statistical Analysis

For the self-esteem and anxiety indicators, an exploratory factor analysis using principal component analysis (PCA) with orthogonal Varimax rotation and Kaiser normalization was performed to reduce collinearity and identify meaningful clusters of items. Factor retention was based on the Kaiser criterion (eigenvalues > 1) and supported by inspection of the Scree plot. Items with cross-loadings were assigned to the component with the highest loading to preserve interpretability of the factor structure. The resulting components were subsequently relabeled using antonyms to reflect low self-esteem and higher anxiety, facilitating interpretation in regression analyses.

- **Self-Esteem Scale:** For the 25-item self-esteem scale (transformed score range 0–100), six components were identified: C1. *Personal Satisfaction* (items 3, 10, 12, 13, 15, 16, 21, 23, 24), C2. *Family understanding* (items 9, 11, 14, 20, 22), C3. *Competence* (items 2, 4, 7), C4. *Social Integration* (items 5, 8, 18, 19), C5. *Self-Affirmation* (items 6, 25), and C6. *Empathy* (items 1, 17). These six factors explained 55.9% of the total variance (KMO = 0.874, *p* = 0.001). To facilitate interpretation for regression modeling, antonyms were applied to label the resulting factors as follows: C1. *Personal Dissatisfaction*, C2. *Family Conflict*, C3. *Incompetence*, C4. *Social Isolation*, C5. *Self-Devaluation* and C6. *Indifference*. The lowest tertile of each component score was used as the dependent variable, reflecting low self-esteem.
- **State Anxiety Scale:** From the 20-item state anxiety scale (scores 20–80), four components were extracted: C1. *Feelings of Unease* (items 2, 5, 10, 11, 16, 19, 20), C2. *Feelings of Exasperation* (items 3, 4, 6, 9, 13, 14, 18), C3. *Feelings of Fear* (items 1, 7, 12, 17), and C4. *Feelings of Fatigue* (items 8, 15). These components explained 50% of the total variance (KMO = 0.898, *p* = 0.001).
- **Trait Anxiety Scale:** For the 20-item trait anxiety scale (scores 20–80), four components were also identified: C1. *Feelings of Vulnerability* (items 21, 26, 27, 30, 33, 36, 39), C2. *Feelings of Restlessness* (items 22, 25, 29, 31, 32, 37, 40), C3. *Feelings of Sadness* (items 23, 24, 28, 38), and C4. *Feelings of Confusion* (items 34, 35). Together, these factors explained 56% of the total variance (KMO = 0.891, *p* = 0.001) (see Supplementary Material S1).

A univariate analysis was conducted to compare categorical and continuous variables using nonparametric tests. Component scores were summed for bivariate analyses. Dummy variables were then created to represent high-risk tertiles (coded as 1) versus low-risk tertiles (coded as 0) for anxiety scales and, conversely, low tertiles (coded as 1) versus high tertiles (coded as 0) for self-esteem components. These binary variables (coded as 1) were included in logistic regression models either as outcomes or exposure variables. The scores for the continuous components of self-esteem and anxiety were categorized into tertiles to facilitate interpretation and comparability in the regression analyses. The lowest tertile of the self-esteem scale was used to represent low self-esteem, and the highest tertile of anxiety was used to identify anxiety risk, thus allowing for the identification of individuals at greater psychological risk. This approach is commonly used in epidemiological and psychosocial research when established clinical cut-off points are unavailable, and it allows for clearer communication of results while reducing the influence of extreme values (Bandeira et al., 2025; Bennette & Vickers, 2012; Yannakoulia et al., 2008).

Logistic regression models were used to assess the associations between sociodemographic variables and mental health indicators (outcomes). Guided by a conceptual causal framework (DAG-based reasoning), sociodemographic variables were modeled as independent exposures rather than confounders, as they represent structural and contextual determinants of mental health. Adjustment was therefore limited to hours of nocturnal sleep to minimize residual confounding while avoiding overadjustment.

Reference categories were defined as follows: education, secondary school or higher; family structure, nuclear or single-parent household; marital status, single or married; socioeconomic status, medium or lower-middle level; occupation, student or homemaker; and age, 15 years or older.

All statistical analyses were conducted using SPSS version 23 and the R statistical software (version 4.4.3).

## 3. Results

### 3.1. Participant Profile

#### 3.1.1. Sociodemographic Characteristics

A total of 338 pregnant adolescents participated in the study. The mean age was 15.9 ± 1.4 years, with 15% being under 15 years old. Participants had a median gynecological age of 4 years (interquartile range [IQR] 3–5) and a median age at menarche of 12 years (IQR 11–12). The mean gestational age at assessment was 25 ± 7 weeks, and prenatal care commenced at a median of 19 weeks (IQR 14.6–25.0) of gestation.

Of the 158 participants who provided their partner’s age data, 59% indicated partners aged over 18 years, with partner ages ranging from 13 to 38 years. Regarding educational attainment, 27% had completed primary school or less, 58% had completed secondary school, and 16% had completed high school. In terms of family composition, 51% came from nuclear families, 20% from extended families, 21% from maternal single-parent households, and 8% resided in composite families (with a stepparent). A significant majority of adolescents (75%) were single, while the remainder lived with a partner. Socioeconomic status varied: 39% of participants were classified as very low, 43% as low, and 18% as lower-middle level. Occupationally, 18% were students, approximately 10% were employed, and 72% were homemakers.

#### 3.1.2. Mental Health Prevalence and Sleep Patterns

Among all participants, 67% reported low self-esteem, 33.5% exhibited depressive symptoms, 52% reported high perceived stress, 18% experienced state anxiety, and 23% had trait anxiety. Regarding sleep patterns, 22% reported 8 to 9 h of sleep, 7% slept 7 h or less, and a substantial 71% reported sleeping more than 10 h per day. A significant majority (87%) typically commenced sleeping late at night.

### 3.2. Associations Between Sociodemographic Factors and Mental Health

As detailed in Table 1, adolescents with a high school education achieved higher scores on the *personal satisfaction* component (C1) of the self-esteem scale (*p* = 0.040) compared to those with lower levels of educational attainment. Adolescents from extended or composite families reported lower scores on the *family understanding* component (C2) than those from nuclear or single-parent families (*p* = 0.001). Furthermore, those from composite families were recorded to report higher levels of perceived stress (*p* = 0.008).

Regarding marital status (see Table 2), partnered adolescents (i.e., those living with a partner or married) exhibited higher depressive symptomatology (*p* = 0.034) and lower self-esteem, particularly in *personal satisfaction* (C1) (*p* = 0.004). For state anxiety, partnered adolescents displayed higher overall state anxiety scores (*p* = 0.050) and specifically elevated scores in *feelings of exasperation* (C2) (*p* = 0.038) and *feelings of fear* (C3) (*p* = 0.040). Similarly, in the trait anxiety dimension, partnered adolescents showed higher overall trait anxiety scores (*p* = 0.024) and greater *feelings of sadness* (C3) (*p* = 0.001) compared with single participants.

With respect to socioeconomic status, adolescents classified in the very low-income group tended to manifest more depressive symptoms (*p* = 0.091), although no other significant differences were observed across the remaining mental health indicators.

Regarding occupation, Table 3 illustrates that employed adolescents scored significantly higher in depressive symptomatology (*p* = 0.001) compared with those who were continuing their studies. They also reported lower self-esteem, specifically in *personal satisfaction* (C1) (*p* = 0.012) and *family understanding* (C2) (*p* = 0.019). Furthermore, working adolescents exhibited higher total scores for both state and trait anxiety, and within the trait anxiety dimension, they showed elevated scores in the *feelings of vulnerability* and *feelings of restlessness* components. Finally, they reported greater perceived stress in response to life situations (*p* = 0.005).

Adolescents under 15 years of age demonstrated significantly lower scores in *self-affirmation* (C5) (*p* = 0.008); no other age categories showed statistically significant differences.

**Figure 1:**
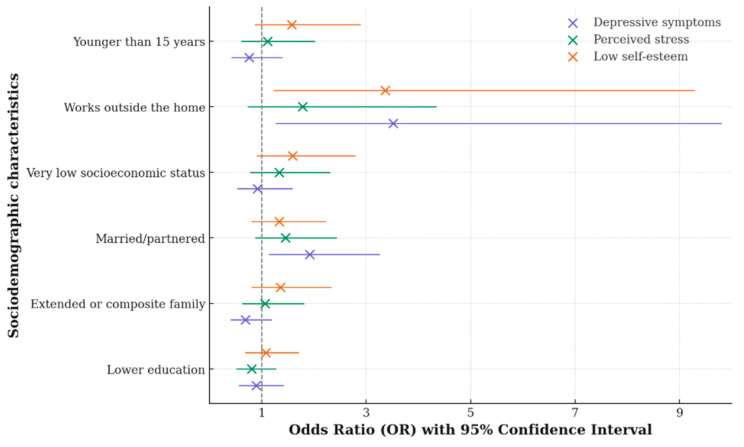
Sociodemographic Variables Associated with Mental Health Risk Indicators.

As depicted in Figure 1, adolescents living with a partner (whether in partnership or married) were more likely to report depressive symptoms (OR 1.921, 95% CI 1.132–3.258, *p* = 0.015). Adolescents employed outside the home were more likely to report depressive symptomatology (OR 3.516, 95% CI 1.260–9.808, *p* = 0.016) and significantly lower self-esteem (OR 3.364, 95% CI 1.219–9.285) compared with those who were studying.

As shown in Table 4, lower educational attainment was associated with a higher likelihood of behavioral manifestations of *personal devaluation* (OR 1.885), as well as belonging to a composite or extended family structure characterized by greater *family conflict* (OR 1.894). Living with a partner (cohabiting/married) was associated with an increased risk of depressive symptomatology (OR 1.921), total state anxiety (OR 1.772), feelings of *exasperation* (OR 1.732), and *sadness* within trait anxiety (OR 2.335). Additionally, adolescents working outside the home had a higher probability of lower overall self-esteem (OR 3.364), greater *personal dissatisfaction* (OR 1.091), increased depressive symptomatology (OR 3.516), state anxiety manifested as restlessness (OR 2.803), and trait anxiety expressed as *sadness* (OR 2.455). Further details are provided in Supplementary Tables S1–S3.

## 4. Discussion

Our findings demonstrate that pregnant adolescents face a substantial burden of mental health problems, primarily characterized by challenges related to self-esteem, depressive symptoms, and anxiety. These challenges do not occur in isolation but rather are closely associated with the complex interplay of multiple sociodemographic factors—encompassing structural elements like education, relational aspects such as marital status and family support, and personal circumstances like occupation.

In this study, key mental health risk indicators—low self-esteem, depressive symptoms, and anxiety—were significantly associated with lower educational attainment, belonging to extended or composite families, living with a partner, and working outside the home. These results are consistent with the systematic review by Mutahi et al. (2022), which identified depressive symptoms as the most frequent mental health concern among pregnant adolescents, with prevalence rates ranging from 10% to over 40% depending on the instrument used (e.g., EPDS, PHQ-9). Notably, the approximately 30% prevalence of depressive symptomatology observed in our cohort is nearly double that reported in a previous Mexican study (deCastro et al., 2011). This finding is concerning, as it suggests a potential deterioration of mental health among pregnant adolescents over time, which could progress to clinically significant disorders such as major depression (Siegel & Brandon, 2014). Consequently, this represents not only an individual health issue but also imposes a considerable economic and social burden on families and the healthcare system (Pi et al., 2025).

In the Mexican context, this heightened vulnerability reflects broader structural inequities and often limited family support networks (Soto-Chavarría et al., 2023), contributing to a progressive emotional deterioration in adolescents (Taşdemir et al., 2010). Our study revealed that over 80% of participants discontinued their education during pregnancy. This pattern severely restricts future opportunities and perpetuates social inequality, particularly in low-resource settings.

We also found that belonging to extended or composite families was associated with lower self-esteem, specifically within the component related to *family conflict*. This likely reflects internal tensions, unclear roles, and unbalanced expectations often present in such household structures. This observation aligns with studies from South Africa and other regions, which highlight how family rejection, social stigma, and insufficient familial support exacerbate depressive symptoms in adolescent mothers (AMAI, 2014; Muthelo et al., 2024). Further research suggests that family functionality is a critical determinant of emotional outcomes and that family-centered interventions can effectively reduce depressive and anxiety symptoms during adolescent pregnancy (Duran et al., 2025). In our cohort, adolescents from nuclear families reported lower stress levels, which is consistent with evidence indicating that extended, composite, or single-parent households often exhibit less organization, less empathetic leadership (Andhavarapu et al., 2021), and less individual attention for family members. Moreover, feelings of loneliness and indifference can emerge during pregnancy regardless of family structure (Sámano et al., 2017), further compounding emotional distress in the short and medium term.

Adolescents living with a partner exhibited a greater likelihood of experiencing depressive symptoms and state anxiety, including feelings of exasperation and sadness. Previous studies indicate that relationship conflict, emotional dependence, poor communication, emotional abuse, and lack of support are significant contributors to these symptoms. Economic vulnerability can further intensify the stress associated with cohabitation (Andhavarapu et al., 2021; Corcoran, 2016; Laurenzi et al., 2020; Sámano et al., 2017). This situation may be exacerbated by a significant age gap between partners. In our study, nearly 60% of partners were over 18 years old, with some reaching 38 years, suggesting that such age disparity could create emotional and communication imbalances within the relationship.

Regarding occupation, our results underscore the critical importance of promoting educational continuity. Early entry into the workforce, particularly when combined with limited schooling, can negatively affect mental health, possibly through impaired decision-making capabilities and heightened feelings of inadequacy (Díaz-Franco, 2007). Working adolescents in our study exhibited higher depressive symptoms and lower self-esteem compared to those who were students or homemakers. Reviews of psychosocial interventions consistently note that economic hardship and precarious employment heighten vulnerability to depression, anxiety, and stress (Andhavarapu et al., 2021; Corcoran, 2016), especially in settings where low education and income correlate with unstable, low-paying jobs and greater uncertainty. This may be related to the higher perceived stress observed among working adolescents in our study, often compounded by complex family arrangements such as extended, composite, or single-parent households, though evidence specific to adolescent pregnancy populations remains scarce (Andhavarapu et al., 2021).

Collectively, our findings suggest that living with a partner, belonging to extended or composite families, low socioeconomic status, and school dropout are closely linked to poorer mental health outcomes. These results underscore the urgent need for comprehensive, non-judgmental care that includes ongoing psychosocial support and clear, age-appropriate information. Adolescents, a group navigating crucial identity formation, require targeted interventions. Qualitative research (Widyaningrum et al., 2025) has already shown that adolescent health behaviors may be compromised during pregnancy, impacting both maternal and infant well-being.

We therefore recommend that pregnant adolescents receive integrated health services encompassing not only obstetric monitoring but also specific interventions aimed at reducing anxiety, fear, exasperation, and sadness. Routine screening for depressive symptoms, anxiety, and self-esteem should be systematically implemented to enable early identification of risks and provision of adequate support (Osok et al., 2018). At the policy level, it is crucial to ensure school retention and develop psychosocial support programs specifically tailored to vulnerable adolescents. Our results strongly support these initiatives, as continuing education and remaining single were associated with lower anxiety and depressive symptoms and higher self-esteem compared to adolescents who entered the workforce prematurely.

Recent European evidence highlights the persistent role of socioeconomic disadvantage, educational disruption, and family context in shaping adolescent pregnancy trajectories and associated health outcomes. A large retrospective cohort study from Southeastern Romania reported that adolescent pregnancy was strongly associated with lower educational attainment, rural residence, and unstable marital circumstances, underscoring the structural vulnerabilities faced by this population (Brezeanu et al., 2025). These findings are consistent with our results and support the interpretation that sociodemographic disadvantage is closely intertwined with adverse psychological outcomes during adolescent pregnancy.

A notable limitation of this study is its cross-sectional design, which precludes the establishment of causal relationships. Nevertheless, to our knowledge, this is the first study in an adolescent population to integrate multiple mental health risk indicators with a comprehensive set of sociodemographic determinants within a highly vulnerable group. A key strength lies in the detailed analysis of the multidimensional components of self-esteem and anxiety, which allowed for a more nuanced understanding of specific emotional vulnerabilities that would not be evident through global scale scores alone. The possible potential temporal variation in psychological outcomes was considered; however, the primary objective of the study was to examine associations rather than temporal trends. All analyses were conducted using harmonized data, and temporal variation is acknowledged as a potential limitation.

A weakness of this study is that partner age was reported for only 158 of the 338 adolescents (less than 50% of the sample), limiting the generalizability of observations regarding partners’ ages. Although the wide range of partner ages (13–38 years) may have ethical and social implications, these data were descriptive and not included in inferential analyses. Prior studies suggest that adolescents with older partners may face distinct psychosocial risks and reduced social support, which could influence mental health outcomes, though evidence remains limited (Agurcia et al., 2001; Sezgin & Punamäki, 2020). Future research with complete partner age data is needed to better understand how partner characteristics affect the mental health of pregnant adolescents.

Our findings robustly indicate that mental health problems during adolescent pregnancy are strongly associated with prevailing sociodemographic conditions. Low education, limited family support, cohabitation, and employment pressures emerge as factors associated with depressive symptoms, anxiety, and low self-esteem. This constellation of factors reflects a state of elevated psychological vulnerability, which, combined with low education and young maternal age, may compromise future economic stability and contribute to a substantial social and public health burden for families and the healthcare system.

Although the study was conducted in a single referral center, the population is representative of pregnant adolescents receiving public-sector perinatal care in urban, low-resource settings, which account for a substantial proportion of adolescent pregnancies in Mexico. Nevertheless, findings may not fully generalize to adolescents attending private or community-based services, who may differ in social, economic, or health characteristics. These results provide important insights into mental health risk patterns in a high-vulnerability urban population and highlight opportunities for future multi-center or population-based studies to evaluate whether these associations are consistent across diverse settings.

## 5. Conclusions

Our study indicates that occupation, family structure, and marital status are closely associated with the mental health outcomes among pregnant adolescents, including depressive symptoms, anxiety, low self-esteem, and perceived stress. These findings suggest that supporting adolescents’ access to education and facilitating informed, autonomous choices regarding cohabitation or marital arrangements may help promote psychological resilience and overall social well-being. Such considerations are particularly relevant in the context of structural inequalities, limited family support, and socioeconomic vulnerability, which can exacerbate emotional distress in this population.

## Figures and Tables

**Table 1:** Scores for Mental Health Risk Indicators According to Educational Level and Family Structure.

	Education				Family Structure				
Variable	Primary, *n* = 90	Secondary, *n* = 194	High School, *n* = 54	*p*	Nuclear, *n* = 172	Extended, *n* = 68	Composite, *n* = 27	Single Parent, *n* = 71	*p*
**Mental health outcomes**									
Depressive symptomatology	9 (4–15)	9 (4–14)	10 (5–15)	0.507	9 (4–13)	8 (4–15)	10 (6–16)	9 (5–17)	0.629
Total self-esteem	72 (52–80)	72 (56–80)	72 (60–80)	0.566	72 (56–80)	70 (44–84)	68 (40–76)	72 (60–80)	0.380
State anxiety	33 (28–38)	32 (28–37)	32 (30–36)	0.464	31 (28–37)	32 (28–37)	34 (30–44)	32 (28–38)	0.269
Trait anxiety	39 (34–47)	38 (31–45)	40 (35–46)	0.183	37 (32–45)	39 (34–45)	43 (38–47)	38 (33–50)	0.311
Perceived stress	7 (5–9)	6 (5–8)	7 (5–8)	0.627	**6 (4–8)**	**7 (5–9)**	**8 (6–10)**	**7 (5–9)**	**0.008**
**Self-esteem components**									
C1 Personal satisfaction	28 (16–32)	28 (16–32)	28 (16–32)	0.907	28 (16–32)	16 (12–20)	22 (12–27)	28 (16–32)	0.052
C2 Family understanding	20 (12–20)	20 (12–20)	20 (16–20)	0.800	**20 (16–20)**	**16 (12–20)**	**16 (8–20)**	**20 (16–20)**	**0.001**
C3 Competence	12 (8–12)	12 (8–12)	12 (8–12)	0.606	12 (8–12)	12 (8–12)	12 (8–12)	12 (8–12)	0.746
C4 Social integration	4 (0–8)	4 (0–8)	4 (0–8)	0.666	4 (0–8)	4 (0–8)	4 (0–8)	4 (0–8)	0.973
C5 Self-affirmation	**4 (4–4)**	**4 (4–8)**	**4 (4–8)**	**0.040**	4 (4–4)	4 (4–8)	4 (4–8)	4 (4–8)	0.479
C6 Empathy	4 (4–8)	4 (4–8)	8 (4–8)	0.675	4 (4–8)	4 (4–8)	4 (4–8)	8 (4–8)	0.973
**State anxiety components**									
C1 Feelings of unease	13 (10–15)	13 (9–15)	14 (11–15)	0.577	12 (9–14)	14 (11–15)	13 (9–16)	14 (10–16)	0.306
C2 Feelings of exasperation	9 (8–12)	9 (8–11)	8 (8–12)	0.935	9 (8–11)	9 (7–11)	9 (8–13)	9 (7–11)	0.262
C3 Feelings of fear	4 (3–6)	5 (3–6)	5 (3–6)	0.705	4 (3–6)	4 (3–6)	5 (4–6)	4 (3–6)	0.197
C4 Feelings of fatigue	6 (4–7)	5 (4–6)	5 (4–6)	0.291	5 (4–6)	6 (4–6)	6 (4–6)	5 (4–7)	0.895
**Trait anxiety components**									
C1 Feelings of vulnerability	13 (11–16)	14 (10–17)	15 (11–17)	0.504	13 (10–16)	14 (12–16)	14 (11–17)	13 (10–17)	0.670
C2 Feelings of restlessness	15 (12–18)	14 (11–18)	16 (13–7)	0.170	14 (12–17)	15 (11–18)	16 (12–18)	15 (12–18)	0.503
C3 Feelings of sadness	6 (5–9)	6 (5–9)	7 (5–9)	0.387	6 (5–8)	7 (5–8)	7 (6–9)	6 (5–10)	0.405
C4 Feelings of confusion	4 (3–5)	3 (3–5)	4 (3–5)	0.427	4 (–5)	3 (3–5)	4 (3–5)	4 (3–5)	0.696

Data are presented as median, 25th–75th percentile; *p*-values were calculated using the Kruskal–Wallis test for comparison of medians. Components correspond to factor-derived subscales; mental health outcomes represent global scale scores. Note: Bold values indicate statistical significance (*p* < 0.05).

**Table 2:** Scores for Mental Health Risk Indicators According to Marital Status and Socioeconomic Level.

	Marital Status			Socioeconomic Level			
Variable	Single, *n* = 260	Partnered/Married, *n* = 78	*p*	Middle, *n* = 60	Low, *n* = 146	Very Low, *n* = 132	*p*
**Mental health outcomes**							
Depressive symptomatology	9 (4–14)	10.5 (6–15)	**0.034**	9 (4–13)	11 (5–15)	9 (4–15)	0.091
Total self-esteem	72 (56–80)	70 (44–80)	0.080	76 (60–84)	68 (48–80)	72 (56–80)	0.271
State anxiety	32 (28–36)	33 (28–41)	**0.050**	31 (28–35)	32 (28–37)	32 (28–37)	0.949
Trait anxiety	37 (32–45)	42 (34–47)	**0.024**	39 (32–47)	39 (33–46)	37 (33–46)	0.496
Perceived stress	7 (5–8)	7 (5–9)	0.077	6 (5–8)	7 (5–9)	7 (5–8)	0.457
**Self-esteem components**							
C1 Personal satisfaction	28 (20–32)	24 (12–28)	**0.004**	28 (20–32)	28 (14–30)	28 (16–32)	0.152
C2 Family understanding	20 (16–20)	20 (12–20)	0.420	20 (16–20)	20 (12–20)	20 (12–20)	0.427
C3 Competence	12 (8–12)	12 (8–12)	0.483	12 (8–20)	12 (8–20)	12 (8–20)	0.754
C4 Social integration	4 (0–8)	4 (0–8)	0.329	4 (4–8)	4 (0–8)	4 (0–8)	0.164
C5 Self-affirmation	4 (4–8)	4 (4–8)	0.479	4 (4–8)	4 (4–4)	4 (4–8)	0.645
C6 Empathy	4 (4–8)	8 (4–8)	0.198	4 (4–8)	4 (4–8)	4 (4–8)	0.700
**State anxiety components**							
C1 Feelings of unease	13 (10–15)	13 (10–16)	0.321	13 (10–15)	13 (9–14)	14 (10–16)	0.218
C2 Feelings of exasperation	9 (8–11)	9 (8–13)	**0.038**	9 (8–10)	9 (8–11)	9 (8–11)	0.837
C3 Feelings of fear	4 (3–6)	5 (4–6)	**0.040**	4 (3–6)	4 (4–6)	5 (3–6)	0.854
C4 Feelings of fatigue	5 (4–6)	6 (4–6)	0.974	6 (4–6)	6 (4–7)	5 (4–6)	0.244
**Trait anxiety components**							
C1 Feelings of vulnerability	13 (10–16)	14 (11–17)	0.114	13 (11–16)	13 (10–17)	14 (10–179	0.687
C2 Feelings of restlessness	14 (11–17)	15 (12–18)	0.410	15 (11–18)	15 (12–18)	14 (12–18)	0.557
C3 Feelings of sadness	6 (5–8)	8 (5–10)	**0.001**	7 (5–9)	7 (5–9)	6 (5–9)	0.177
C4 Feelings of confusion	4 (3–5)	4 (3–5)	0.367	3 (3–4)	4 (3–5)	4 (3–5)	0.974

Data are presented as median, 25th–75th percentile; *p*-values calculated using the Kruskal–Wallis test. Components correspond to factor-derived subscales; mental health outcomes represent global scale scores. Note: Bold values indicate statistical significance (*p* < 0.05).

**Table 3:** Scores for Mental Health Risk Indicators According to Occupation and Age.

	Occupation				Age		
Variable	Home, *n* = 242	Student, *n* = 59	Employed, *n* = 37	*p*	<15 years, *n* = 53	≥15 years, *n* = 285	*p*
**Mental health outcomes**							
Depressive symptomatology	9 (5–14)	6 (2–1)	14 (10–19)	**0.001**	8 (4–14)	10 (5–14)	0.950
Total self-esteem	72 (56–80)	76 (64–84)	58 (36–78)	**0.012**	68 (52–80)	72 (56–80)	0.327
State anxiety	32 (28–37)	21 (27–36)	35 (30–47)	**0.038**	32 (26–37)	32 (28–37)	0.181
Trait anxiety	39 (33–45)	35 (31–44)	46 (11–17)	**0.020**	40 (33–47)	38 (32–46)	0.814
Perceived stress	7 (5–9)	5 (4–7)	8 (6–9)	**0.005**	7 (5–9)	7 (5–9)	0.439
**Self-esteem components**							
C1 Personal satisfaction	28 (16–32)	28 (24–32)	16 (4–32)	**0.019**	28 (12–20)	28 (16–32)	0.513
C2 Family understanding	20 (12–20)	20 (16–20)	20 (12–20)	0.139	20 (16–20)	20 (12–20)	0.704
C3 Competence	12 (8–12)	12 (8–12)	12 (6–12)	0.258	12 (8–12)	12 (8–12)	0.284
C4 Social integration	4 (0–8)	4 (4–12)	4 (0–6)	0.078	4 (0–8)	4 (0–8)	0.270
C5 Self-affirmation	4 (4–8)	4 (4–8)	4 (4–4)	0.326	**4 (4–4)**	**4 (4–8)**	**0.008**
C6 Empathy	4 (4–8)	4 (4–8)	8 (4–8)	0.909	4 (4–8)	4 (4–8)	0.210
**State anxiety components**							
C1 Feelings of unease	13 (10–15)	12 (9–14)	14 (13–17)	0.085	12 (10–14)	13 (10–15)	0.085
C2 Feelings of exasperation	9 (7–11)	8 (8–10)	10 (8–15)	0.151	8 (8–11)	9 (8–11)	0.760
C3 Feelings of fear	5 (3–6)	4 (3–5)	6 (3–89	0.251	4 (3–6)	5 (3–6)	0.204
C4 Feelings of fatigue	5 (4–6)	6 (4–6)	6 (5–6)	0.622	5 (4–6)	6 (4–6)	0.421
**Trait anxiety components**							
C1 Feelings of vulnerability	14 (11–17)	12 (9–16)	16 (11–20)	**0.029**	13 (9–15)	14 (10–17)	0.605
C2 Feelings of restlessness	15 (12–18)	13 (10–17)	17 (13–21)	**0.034**	15 (12–18)	14 (12–18)	0.388
C3 Feelings of sadness	6 (5–9)	6 (5–9)	8 (6–11)	0.155	6 (4–9)	6 (5–9)	0.589
C4 Feelings of confusion	4 (3–5)	4 (3–5)	4 (3–5)	0.982	4 (3–5)	4 (3–5)	0.391

Data are presented as median, 25th–75th percentile; *p*-values were obtained using the Kruskal–Wallis test for comparison of medians. Note: Bold values indicate statistical significance (*p* < 0.05).

**Table 4:** Logistic regression of sociodemographic factors associated with mental health outcomes and factor-derived components.

Sociodemographic Factor	Outcome/Component	OR (95% CI)
**Educational level ^1^**		
Lower educational attainment	Self-esteem—*Self-Devaluation* (component)	1.885 (1.00–3.540)
**Family structure ^2^**		
Extended/composite	Self-esteem—*Family conflicts* (component)	1.894 (1.093–3.265)
**Marital status ^3^**		
Partnered/married	Depressive symptomatology (total score)	1.921 (1.132–3.258)
	State anxiety—Exasperation (component)	1.732 (1.022–2.937)
	State anxiety (total score)	1.772 (1.049–2.993)
	Trait anxiety—Sadness (component)	2.335 (1.382–3.944)
**Occupation ^4^**		
Employed	Self-esteem (total score)	3.364 (1.290–9.285)
	Self-esteem—Personal dissatisfaction (component)	1.091 (1.072–6.646)
	Depressive symptomatology (total score)	3.516 (1.260–9.808)
	State anxiety—Restlessness (component)	2.803 (1.078–7.287)
	Trait anxiety—Sadness (component)	2.455 (1.009–6.053)

Odds ratios (OR) were obtained from logistic regression models adjusted for hours of sleep. Outcomes correspond to global scale scores. Components correspond to factor-derived subscales obtained through exploratory factor analysis. Reference categories: ^1^ High school education or higher; ^2^ Nuclear or single-parent family; ^3^ Single marital status; ^4^ Student or homemaker.

## Data Availability

Additional data and information supporting the reported results are available upon request from the corresponding author.

## Supplementary Materials

The following supporting information can be downloaded at: https://www.mdpi.com/article/10.3390/bs16020221/s1, Supplementary Material S1: Exploratory component analysis (PCA): factor loadings, variance explained, and analytic decisions. Table S1: Logistic regression models for risk components associated with low self-esteem. Table S2: Binary logistic regression models for risk components of state anxiety symptomatology. Table S3: Binary logistic regression models for risk components of trait anxiety symptomatology.

## Abbreviations

The following abbreviations are used in this manuscript:

EPDS	Edinburgh Postnatal Depression Scale
STAI	State–trait anxiety Inventory
PSS-4	Perceived Stress Scale
ENADID	National Survey on Demographic Dynamics
AMAI	Mexican Association of Market Intelligence and Public Opinion Agencies
INPer	Instituto Nacional de Perinatología
OR	Odds ratio
