# Supplementary material for: Mental Health in Pregnant Adolescents: Associations with Family Structure, Educational Continuity, and Marital Status
Source: Behav Sci (Basel). 2026 Feb 3;16(2):221. doi: 10.3390/bs16020221 (PMC12937784; doi:10.3390/bs16020221)
Supplement: Supplementary file 1 [file behavsci-16-00221-s001.zip › Supplementary material S1.pdf]

Supplementary Material S1. Exploratory Component Analysis (PCA): Factor Loadings, Variance Explained, and Analytic Decisions

Components were retained based on eigenvalues >1, interpretability, and consistency with the theoretical structure of each scale.

TRAIT ANXIETY

KMO and Bartlett's Test

Kaiser–Meyer–Olkin measure of sampling adequacy		.898
Bartlett's test of sphericity	Approx. Chi-square	2490.664
	Df (degrees of freedom)	190
	Sig.	.000

Rotated Component Matrix[a]

	Component			
	1	2	3	4
1 I feel calm	.411	.058	.464	.340
2 I feel secure	.664	.187	.287	.040
3 I feel tense	.086	.614	.193	.069
4 I feel upset	.210	.432	.310	-.106
5 I feel at ease	.737	.034	.132	-.022
6 I feel strained	.125	.709	.182	-.023
7 I am presently worrying over possible….	.114	.291	.748	.040
8 I feel rested	.175	-.069	.010	.798
9 I feel anxious	.105	.565	.356	.055
10 I feel comfortable	.707	.003	.215	.120
11 I feel self-assured	.740	.196	-.040	.022
12 I feel nervous	.131	.440	.575	-.131
13 I feel jittery	.048	.813	-.036	.047
14 I feel like I am about to burst	.064	.647	.149	-.005
15 I feel relaxed	.180	.114	.027	.791
16 I feel satisfied	.629	.069	.173	.331

17 I feel worried	.207	.385	.741	.098
18 I feel confused and upset	.142	.756	.200	.059
19 I feel joyful	.707	.091	.057	.186
20 I feel good	.715	.193	.020	.118

Extraction method: Principal component analysis.

Rotation method: Varimax with Kaiser normalization.

a. Rotation converged in 6 iterations.

TRAIT ANXIETY

Total variance explained

	Initial eigenvalues			Extraction sums of squared loadings			Rotation sums of squared loadings		
Component	Total	% of variance	Cumulative %	Total	% of variance	Cumulative %	Total	% of variance	Cumulative %
1	6.227	31.133	31.133	6.227	31.133	31.133	3.848	19.239	19.239
2	2.780	13.900	45.033	2.780	13.900	45.033	3.608	18.041	37.280
3	1.224	6.119	51.152	1.224	6.119	51.152	2.194	10.968	48.248
4	1.027	5.136	56.288	1.027	5.136	56.288	1.608	8.039	56.288

Extraction method: Principal component analysis.

KMO and Bartlett's Test

Kaiser–Meyer–Olkin measure of sampling adequacy		.891
Bartlett's test of sphericity	Approx. Chi-square	1848.537
	Df.	190
	Sig.	.000

STATE ANXIETY

Total variance explained

	Initial eigenvalues			Extraction sums of squared loadings			Rotation sums of squared loadings		
Component	Total	% of variance	Cumulative %	Total	% of variance	Cumulative %	Total	% of variance	Cumulative %
1	5.808	29.038	29.038	5.808	29.038	29.038	3.148	15.739	15.739
2	1.912	9.560	38.599	1.912	9.560	38.599	3.051	15.253	30.992
3	1.182	5.910	44.509	1.182	5.910	44.509	2.438	12.191	43.182
4	1.078	5.388	49.896	1.078	5.388	49.896	1.343	6.714	49.896

Extraction method: Principal component analysis.

STATE ANXIETY

Rotated Component Matrix[a]

	Component			
	1	2	3	4
21 I feel bad	.586	.138	.281	.067
22 I get tired easily	.147	.506	-.168	.265
23 I feel like crying	.247	.206	.541	.311
24 I wish I were as happy as other …	.182	.148	.672	.031
25 I miss opportunities…	.073	.645	.197	.047
26 I feel rested	.551	.034	-.310	.300
27 I am a calm, serene…	.590	.247	-.011	-.216
28 I feel that difficulties pile up to the point that …	.107	.342	.596	.234
29 I worry too much about unimportant…	.108	.691	.135	.118
30 I am happy.	.599	.029	.479	-.010
31 I take things too personally.	.143	.507	.105	.299
32 I lack self-confidence.	.344	.538	.312	.006
33 I feel secure.	.669	.225	.207	.002

34 I try to avoid crises and difficulties	-.186	.162	-.007	.642
35 I feel melancholic	.139	.119	.372	.639
36 I feel satisfied	.712	.047	.104	.084
37 Some unimportant ideas run through my mind.	.025	.631	.346	-.129
38 Disappointments affect me so much that …	.135	.431	.581	-.064
39 I am a stable person.	.651	.173	.186	-.082
40 When I think about the matters I have at hand,	.247	.620	.239	.133

Extraction method: Principal component analysis.
Rotation method: Varimax with Kaiser normalization.
a. Rotation converged in 10 iterations.

SELF-ESTEEM

KMO and Bartlett's Test

Kaiser–Meyer–Olkin measure of sampling adequacy		.874
Bartlett's test of sphericity	Approx. Chi-square	2217.722
	Df.	300
	Sig.	.000

Total variance explained

Component	Initial eigenvalues		Extraction sums of squared loadings			Rotation sums of squared loadings		
	% of variance	Cumulative %	Total	% of variance	Cumulative %	Total	% of variance	Cumulativ e %
1	24.980	24.980	6.245	24.980	24.980	4.160	16.639	16.639
2	6.483	31.463	1.621	6.483	31.463	2.647	10.589	27.228
3	5.820	37.283	1.455	5.820	37.283	1.806	7.222	34.450
4	5.267	42.551	1.317	5.267	42.551	1.664	6.654	41.105

5	4.736	47.287	1.184	4.736	47.287	1.349	5.396	46.500
6	4.126	55.875	1.032	4.126	55.875	1.171	4.689	55.875

Extraction method: Principal component analysis.

SELF-ESTEEM

Rotated Component Matrix[a]

	Component					
	1	2	3	4	5	6
1.Problems affect me very little.	.040	-.049	.039	.041	-.147	.842
2.I find it difficult to speak in public.	.111	-.015	.653	.212	.027	.003
3.I would change things about myself.	.450	.158	.324	.084	.157	.142
4.I make decisions easily.	.241	.076	.531	.158	.312	-.102
5.I am friendly.	.044	.162	.050	.525	.353	-.003
6.I get angry easily.	.196	.105	.157	.066	.664	.253
7.I find it hard to adapt to something new.	.104	.096	.741	-.103	-.113	.063
8.I am popular.	-.170	.077	.301	.584	-.166	.142
9.My feelings matter.	.088	.775	.115	.140	.095	-.104
10.I give up easily.	.455	.167	.384	.016	.128	-.206
11.My family expects a lot from me.	-.022	-.069	-.014	.040	.069	.867
12. I find it difficult to accept myself as I am	.740	.040	.127	.184	.136	-.153
13.My life is complicated.	.656	.304	.080	-.084	-.068	.004
14.My ideas are almost always accepted.	.127	.453	.097	.392	-.116	.102
15.I have a poor opinion of myself.	.764	.105	.054	.222	.035	-.006
16.I would like to leave my home.	.469	.533	-.005	-.067	-.038	.014
17.I dislike my work.	.248	.215	.017	.079	.351	.488
18.I am less attractive.	.400	.007	-.009	.545	.048	.023
19.If I have something to say, I say it.	.373	.054	.028	.451	.072	-.165
20.My family understands me.	.173	.779	.048	.189	.059	-.119
21.Others are more accepted than I am.	.431	.412	-.030	.306	-.195	-.255

22.My family puts pressure on me.	.341	.628	.046	-.180	.209	.182
23.I feel encouraged by what I do.	.626	.183	.297	.082	.218	-.004
24.I would like to be another person.	.704	.263	.106	.030	-.033	.096
25.I am not very trustworthy.	.462	.106	.151	.080	-.514	.250

Extraction method: Principal component analysis.

Rotation method: Varimax with Kaiser normalization.

a. Rotation converged in 15 iterations.
