# Supplementary material for: Mental Health in Pregnant Adolescents: Associations with Family Structure, Educational Continuity, and Marital Status
Source: Behav Sci (Basel). 2026 Feb 3;16(2):221. doi: 10.3390/bs16020221 (PMC12937784; doi:10.3390/bs16020221)
Supplement: Supplementary file 1 [file behavsci-16-00221-s001.zip › Tables S1-S3.pdf]

Supplementary Table S1. Logistic regression by risk components for lower self-esteem

	Personal dissatisfaction (OR, 95% CI)	Family conflicts (OR, 95% CI)	Incompetence (OR, 95% CI)	Social isolation (OR, 95% CI)	Self-devaluation (OR, 95% CI)	Indifference (OR, 95% CI)
Lower educational level[1]	0.986 (0.611–1.592)	**1.089 (0.674–1.759)**	0.823 (0.507–1.334)	0.867 (0.542–1.387)	**1.885 (1.003–3.540)**	1.249 (0.792–1.968)
Extended or composite family[2]	1.073 (0.897–1.284)	1.894 (1.093–3.265)	1.242 (0.709–2.173)	1.051 (0.607–1.819)	0.642 (0.363–1.135)	1.022 (0.601–1.740)
Married/with partner[3]	1.563 (0.566–4.312)	0.578 (0.206–1.626)	0.807 (0.466–1.400)	1.252 (0.740–2.118)	1.051 (0.597–1.849)	0.785 (0.473–1.301)
Very low socioeconomic level[4]	1.215 (0.844–1.749)	1.045 (0.727–1.503)	1.406 (0.769–2.573)	1.042 (0.597–1.818)	1.591 (0.891–2.839)	0.959 (0.556–1.654)
Works outside the home[5]	**1.091 (1.072–6.646)**	0.918 (0.438–1.927)	1.499 (0.631–3.565)	1.883 (0.718–4.941)	1.368 (0.485–3.857)	0.798 (0.339–1.876)
Younger than 15 years[6]	1.316 (0.627–2.762)	1.124 (0.934–1.993)	1.703 (0.923–2.573)	1.477 (0.782–2.791)	1.998 (0.921–4.333)	1.336 (0.727–2.454)

[1]Reference: high school education or higher; [2]Reference: nuclear or single-parent family; [3]Reference: single; [4]Reference: middle-low and low socioeconomic level; [5]Reference: being a student; [6]Reference: older than 15 years.
OR (95% CI): Odds ratio (95% confidence interval).

Supplementary Table S2. Logistic regression models for State-Anxiety symptomatology

	Restlessness (OR, 95% CI)	Exasperation (OR, 95% CI)	Fear (OR, 95% CI)	Fatigue (OR, 95% CI)	Total (OR, 95% CI)
Lower educational level[1]	0.898 (0.567–1.424)	1.231 (0.775–1.955)	1.195 (0.757–1.888)	0.968 (0.614–1.526)	1.216 (0.767–1.929)
Extended or composite family[2]	1.334 (0.776–2.295)	0.649 (0.378–1.115)	0.979 (0.574–1.670)	1.117 (0.657–1.901)	1.233 (0.715–2.126)
Married/with partner[3]	1.177 (0.706–1.964)	**1.732 (1.022–2.937)**	1.460 (0.877–2.430)	1.025 (0.618–1.698)	**1.772 (1.049–2.993)**
Very low socioeconomic level[4]	1.138 (0.658–1.967)	1.065 (0.613–1.848)	1.318 (0.762–2.281)	0.891 (0.517–1.536)	1.449 (0.831–2.511)
Works outside the home[5]	**2.803 (1.078–7.287)**	1.970 (0.781–4.972)	1.384 (0.585–3.277)	1.575 (0.658–3.771)	2.101 (0.831–5.309)
Younger than 15 years[6]	0.646 (0.353–1.184)	0.696 (0.380–1.275)	0.681 (0.371–1.248)	0.661 (0.364–1.220)	0.874 (0.478–1.599)

[1]Reference: high school education or higher; [2]Reference: nuclear or single-parent family; [3]Reference: single; [4]Reference: middle-low and low socioeconomic level; [5]Reference: being a student; [6]Reference: older than 15 years.
OR (95% CI): Odds ratio (95% confidence interval).

Supplementary Table S3. Logistic regression model for Trait-Anxiety symptomatology

	Insecurity (OR, 95% CI)	Uneasiness (OR, 95% CI)	Sadness (OR, 95% CI)	Confusion (OR, 95% CI)	Total (OR, 95% CI)
Lower educational level[1]	0.949 (0.596–1.510)	1.162 (0.735–1.838)	0.717 (0.450–1.143)	1.005 (0.636–1.589)	0.902 (0.510–1.595)
Extended or composite family[2]	0.582 (0.496–1.482)	1.256 (0.736–2.144)	1.169 (0.679–2.013)	0.793 (0.465–1.355)	1.303 (0.648–2.618)
Married/with partner[3]	**1.606 (0.964–2.677)**	1.199 (0.721–1.994)	**2.335 (1.382–3.944)**	1.285 (0.771–2.142)	1.522 (0.775–2.991)
Very low socioeconomic level[4]	1.365 (0.773–2.411)	0.819 (0.474–1.415)	0.694 (0.398–1.212)	1.314 (0.762–2.264)	1.242 (0.644–2.393)
Works outside the home[5]	**2.168 (0.913–5.150)**	2.111 (0.861–5.176)	**2.455 (1.009–6.053)**	0.777 (0.331–1.826)	0.933 (0.331–2.634)
Younger than 15 years[6]	0.742 (0.395–1.394)	1.696 (0.921–3.124)	1.167 (0.632–2.155)	0.874 (0.481–1.591)	1.199 (0.561–2.563)

[1]Reference: high school education or higher; [2]Reference: nuclear or single-parent family; [3]Reference: single; [4]Reference: middle-low and low socioeconomic level; [5]Reference: being a student; [6]Reference: older than 15 years.
OR (95% CI): Odds ratio (95% confidence interval).
